# Outcomes of Patients with Pulmonary Large Cell Neuroendocrine Carcinoma in I–IV Stage

**DOI:** 10.3390/medicina57020118

**Published:** 2021-01-28

**Authors:** Anna Lowczak, Agnieszka Kolasinska-Cwikla, Karolina Osowiecka, Lidia Glinka, Jakub Palucki, Robert Rzepko, Anna Doboszynska, Jaroslaw B. Cwikla

**Affiliations:** 1 Department of Pulmonology, Faculty of Medicine, University of Warmia and Mazury in Olsztyn, Jagiellonska 78, 11-041 Olsztyn, Poland; anna.doboszynska@wp.pl; 2 Department of Oncology and Radiotherapy, Maria Sklodowska-Curie Memorial Cancer Center and Institute of Oncology, Warsaw, Poland Roentgena 5, 02-781 Warsaw, Poland; adkolasinska@yahoo.com; 3 Department of Psychology and Sociology of Health and Public Health, School of Public Health, University of Warmia and Mazury in Olsztyn, Warszawska 30, 11-041 Olsztyn, Poland; karolina.osowiecka@uwm.edu.pl; 4 Department of Anesthesiology and Intensive Care Faculty of Medicine, University of Warmia and Mazury in Olsztyn, Warszawska 30, 10-082 Olsztyn, Poland; lidka.glinka@gmail.com; 5 Department of Radiology, Maria Sklodowska-Curie Institute of Oncology in Warsaw, Roentgena 5, 02-781 Warsaw, Poland; jmpalucki@gmail.com; 6 Specialist Hospital in Prabuty, Kuracyjna 30, 82-550 Prabuty, Poland; robert.prabuty@gmail.com; 7 Department of Cardiology and Internal Medicine Faculty of Medicine, University of Warmia and Mazury in Olsztyn, Warszawska 30, 10-082 Olsztyn, Poland; jbcwikla@interia.pl

**Keywords:** pulmonary large cell neuroendocrine cancer, overall survival, progression-free survival

## Abstract

*Background and Objectives*: Large cell neuroendocrine cancer is characterised by poor prognosis. The standard of treatment is still not established. The aim of this study was to assess the predictive factors of overall survival (OS) and progression-free survival (PFS) of pulmonary large cell neuroendocrine carcinoma (LCNEC) and combined LCNEC. *Materials and Methods*: All patients had confirmed pathology stage I-IV disease recorded between period 2002–2018. Survival curves were estimated by Kaplan–Meier method. Uni- and multivariable analysis was conducted using Cox-regression analysis. *Results*: A total of 132 patients with LCNEC and combined LCNEC were included. Half of them had clinical stage IIIB/C-IV. Patients were treated with radical (*n* = 67, including surgery alone; resection with neo-adjuvant or adjuvant chemotherapy, radiochemotherapy, or adjuvant radiotherapy; patients treated with radiochemotherapy alone), palliative (*n* = 41) or symptomatic (*n* = 24) intention. Seventeen patients were treated with resection margin R1 or R2. Non-small cell carcinoma (NSCLC) chemotherapy (platinum-vinorelbine; PN schedule) and small-cell lung carcinoma (SCLC) chemotherapy approaches (platinum/carboplatinum-etoposide; PE/KE schedule) were administered in 20 and in 55 patients, respectively. The median (95% Confidence Interval (CI)) OS and PFS were 17 months (9.0–36.2 months) and 7 months (3.0–15.0 months), respectively. Patients treated with negative resection margin, with lower clinical stage, without lymph node metastasis, and with size of primary tumour ≤4 cm showed significantly better OS and PFS. The main risk factors with an adverse effect on survival were advanced CS and positive resection margin. *Conclusions*: Patients with LCNEC characterized poor prognosis. Independent prognostic factors influencing PFS were initial clinical stage and resection margin R0 vs. R1-2.

## 1. Introduction

Neuroendocrine tumours of the lung are a diverse group of malignant neoplasms in terms of morphology, clinical characteristics, and etiology and represent 20% of all lung cancers [1]. According to WHO classification (2015), four main types are distinguished—typical carcinoids, atypical carcinoids (low-grade tumours), small-cell lung carcinoma (SCLC), and large-cell neuroendocrine carcinoma (LCNEC)—all of which represent a group of high-grade malignant tumours. The current WHO classification defines LCNEC as morphologically non-small cell carcinoma (NSCLC) with histopathological features of neuroendocrine cancer and immunohistochemical expression of neuroendocrine markers. In addition, a subgroup of LCNEC has been distinguished and is defined as LCNEC with components of adenocarcinoma, squamous cell carcinoma, or spindle cell carcinoma, and/or giant-cell carcinoma. Clinical LCNEC behaves biologically aggressively, similarly to SCLC [2,3].

Pulmonary LCNECs are rare tumours of the lung and the incidence appears to be approximately 3% of all lung cancers [4]. The survival prognosis in terms of overall survival (OS) and progression-free survival (PFS) is poor, similar to SCLC. Both, present a high rate of lymph node involvement (60–80%) and presence of distant metastasis (40%) at the initial diagnosis [5]. Five-year survival rates range from 15% to 57% for patients with LCNEC and 30% for patients with combined LCNEC [4,5,6,7]. The poor prognosis is seen even in patients with potentially resectable stage I disease in whom 5-year survival is noted between 27% and 67% of subjects. High incidence of recurrence after radical surgery is observed and develops within the first 2 years of follow-up [8,9,10]. LCNEC, like SCLC, is often correlated with male sex, older age (median 65 years), and heavy smoking habits [5].

There is a lack of prospective randomized trials regarding pulmonary LCNEC due to its rarity and difficulties in the initial diagnosis. Standard of treatment of LCNEC is still not established and data are based primarily on retrospective analysis. The basic method of treatment of LCNEC patients at early stages is radical surgery (most frequently lobectomy and less frequently pneumonectomy with mediastinal lymph node dissection). The criteria of patient’s qualification to surgery treatment and neoadjuvant chemotherapy are the same as NSCLC. The schedule of chemotherapy is still not established, independently of clinical stage of LCNEC. Patients are treated according to small-cell lung cancer (SCLC) schedule of treatment: platinum-based + etoposide, or for NSCLC, platinum + gemcitabine vs. vinorelbine vs. paclitaxel. Although the optimal schedule of adjuvant chemotherapy has not been defined yet, it seems to be beneficial and rational to use in each case of LCNEC [8,11]. Neoadjuvant platinum-based regimens may be an option for patients potentially with initially resectable tumours [12].

The aim of this retrospective study was to estimate the survival in terms of PFS and OS of patients with pulmonary large-cell neuroendocrine carcinoma (LCNEC) and combined LCNEC and identify prognostic factors for OS and PFS in this group of rare lung cancers.

## 2. Materials and Methods

### 2.1. Data Sources

All patients with a pathological diagnosis of stage I-IV large cell neuroendocrine lung cancer (LCNEC) and combined LCNEC, recorded in 3 oncological centres located in central and northeastern regions of Poland between 1 January 2002 and 31 December 2018 were included. Patients included in the analysis met the criteria in Table 1. Analyzed group of patients received radical, palliative, or only symptomatic treatment.

All 132 patients received their diagnosis of cancer based on histopathological examination of tumour sample obtained during core needle biopsy, mediastinoscopy, wedge resection, radical surgery with intention to treat (ITT), or non-radical surgery, as well as tumour sampling or lymph nodes resection. The immunohistochemical methods were used in accordance with WHO recommendations.

The clinical stage of disease was determined using UICC (The Union for International Cancer Control) TNM (T—tumour, N—nodes, M—metastasis) classification of Malignant Tumors—8th edition [13]. The degree of pathological stage of disease (pTNM) was assessed in 60 patients who had surgery with ITT, which was in 45% of subjects in the total population. The degree of clinical stage (cTNM) was assessed in 72 patients (55%) based on the imaging examinations (including PET, CT, MRI, and bone scintigraphy) and tumour biopsy, including endobronchial ultrasound (EBUS) or mediastinoscopy or peripheral lymph node excisional biopsy. The clinical stage (cTNM) was used in case if patients were disqualified from radical surgery due to advanced disease, contraindications to surgery, or concomitant severe other disease, or no patient consent for treatment.

The analysis was retrospective. The data were collected by medical records, hospital databases and registry office and supplemented by interviews with patients, their families, and the attending physician.

The study was conducted in accordance with the Declaration of Helsinki. On 12 April 2018, the protocol was approved by the Ethics Committee of the University of Warmia and Mazury in Olsztyn (12/2018).

### 2.2. Statistical Analysis

Survival probabilities were estimated by Kaplan–Meier method, and differences in survival were compared by the log-rank test. Uni- and multivariable predictors of overall mortality were estimated through Cox-regression analysis. Univariate variables with *p*-value <0.1 were included in the multivariable model. Overall survival (OS) was defined as the time from histopathological diagnosis till death from any cause or last follow-up censored. Progression-free survival (PFS) was calculated from the date of beginning treatment until the first evidence of progression, death, or last day of follow-up. A *p*-value of < 0.05 was considered to be significant. The analysis was conducted using TIBCO Software Inc. (2017). Statistica (data analysis software system), version 13 (StatSoft, Krakow, Poland). http://statistica.io.

## 3. Results

### 3.1. Characteristic of Patients

A total of 132 patients with LCNEC (*n* = 124) and combined LCNEC (*n* = 8) were included in the analysis. Between 2002 and 2018, patients were treated with radical, palliative, or symptomatic intension, in central and northeastern centres in Poland. The follow-up ranged from 0 to 192 months. The mean age of patients was 64.1 and standard deviation (SD) 9.1 years (range: 36–86 years). The group of patients consisted of 47 women (36%) and 85 men (64%). The ratio of women to men was 1:1.81. Half of the patients were in IIIB/C-IV clinical stage, Ki67 > 55, and most patients (71%) had lymph node metastasis and size of tumour >4 cm (62%). The mean size of tumour was 52.2 mm (SD 26.5; range 7–136 mm). Radical treatment was administrated in 51% of patients, including surgery alone (45%), resection with neo- or adjuvant chemotherapy, radiochemotherapy or adjuvant radiotherapy (45%), or radiochemotherapy (10%). In seventeen patients (28% of patients underwent resection) non-radical surgery R1-2 (positive margin) was observed. NSCLC chemotherapy (platinum-vinorelbine; PN schedule) and SCLC chemotherapy (platinum/carboplatinum-etoposide; PE/KE schedule) was administered in 26% and in 71.4% of patients, respectively (Table 2).

### 3.2. Comparison of Outcome

**Figure 1:**
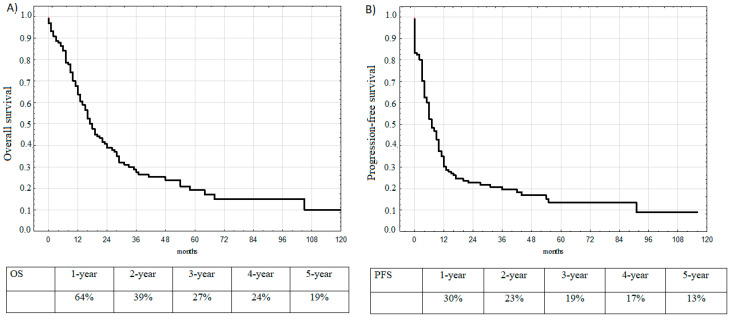
(**A**) Overall survival (OS) of all patients. (**B**) Progression-free survival (PFS) of all patients.

Nintey-seven patients died during the follow-up period. Five-year OS for all patients was 19%. The median overall survival (mOS) was 17 months (95% Confidence Interval (CI): 9.0–36.2 months; Figure 1A). Progression during the observation interval was documented in 107 cases. The median progression-free survival (mPFS) was 7 months (95% CI: 3.0–15.0 months), and 13% of patients survived 5 years without progression (Figure 1B).

Patients treated with radical intension, R0 resection margin, with lower clinical stage (CS), lymph node-negative, and size of tumour ≤4 cm showed significantly better survival prognosis (OS and PFS) (*p* < 0.05). Five-year PFS for patients with R0 vs. R1-2 resection margin was 40% vs. 7%. The median PFS (mPFS) was 31 months (95% CI: 7.0—not reached months) and 9 months (95% CI: 3.0–18.7 months) for R0 vs. R1-2, respectively (*p* = 0.02). According to clinical stage, 5-year OS was 63%, 25%, and 18% respectively for I, II, and III CS, and 5-year PFS was 49% and 8% for I and III CS. The mOS was 105 months (95% CI: 25.6—not reached months) for stage I, 28 months (95% CI: 14.5–53.9 months) for stage II, 23 months (95% CI: 12.4–39.8 months) for stage III, and 8 months (95% CI: 3.0–16.0 months) for stage IV, whereas mPFS was 54 months (95% CI: 6.0-not reached months), 27 months (95% CI: 8.5–40.5 months), 9 months (95% CI: 5.0–17.2 months), and 3 months (95% CI: 0.0–7.0 months), respectively, for I, II, III, and IV CS (*p* < 0.001). Patients with N0 status survived longer time without progression, such that mPFS was 42 months (95% CI: 7.0-not reached) for N0 patients compared to lymph node metastasis patients with mPFS of 5 months (95% CI: 1.3–10.6 months). The mOS was 13 months (95% CI: 7.0–25.8 months) and 64 months (95% CI: 21.9-not-reached) for N+ vs. N−, respectively (*p* < 0.001). Patients with tumour ≤4 cm had significantly better prognosis than patients who received therapy on tumour >4 cm (respectively: 29 months mOS (95% CI: 14.9–81.7 months) vs. 13 months mOS (95% CI: 6.4–28.0) and 10 months mPFS (95% CI: 5.0–46.1 months) vs. 5 months mPFS (95% CI: 1.3–12.0 months; Table 3).

In the univariate model analysis, significant influence on OS was demonstrated for clinical stage, lymph nodes status, size of tumour, intention of treatment, and extent of resection (*p* < 0.05). Patients diagnosed with clinical stage IIIC had about 14 times higher risk of death (HR: 14.4; 95% CI: 3.44–60.27), and patients with CS IV had almost 16 times higher (HR: 15.9; 95% CI: 6.48–39.02). Lymph node metastasis status was found to be significant correlated with higher overall mortality (N0 vs. N3, HR: 9.42; (95% CI: 4.61–19.28). The positive resection margin and large size of tumour significantly increased the percentage of deaths (R0 vs. R1-2, HR: 2.28; 95% CI: 1.12–4.63) and (≤3 cm vs. >7 cm, HR: 4.35; 95% CI: 2.29–8.24), respectively (Table 4). In univariate analysis, a significant influence on PFS was also demonstrated for clinical stage, lymph node status, size of tumour, intention of treatment and extent of resection (*p* < 0.05) (Table 4).

In the multivariate analysis, it was estimated that clinical stage was an independent factor influencing OS (I vs. II, HR: 4.0; 95% CI: 1.43–11.16; *p* = 0.008). The risk of progression increased in more advanced clinical stage (I vs. IV, HR: 10.53; 95% CI: 2.29–48.38; *p* = 0.002) and positive resection margin (R0 vs. R1-2, HR: 3.06; 95% CI: 1.26–7.42; *p* = 0.01) (Table 5).

## 4. Discussion

Large cell neuroendocrine carcinoma (LCNEC) is a rare but aggressive type of lung cancer, with an incidence of about 3% [14]. We reported 132 patients diagnosed with LCNEC during 17 years in daily practice, on average 64 years of age, most of whom were male (64%). Almost half of them had advanced clinical stage, 40% of patients (*n* = 53) with stage IV. Similarly, Naidoo J. [15] also reported a large series of LCNEC patients in stage IV, 49 patients during 8 years, 64 years in age and 63% of patients of whom were male. In the present study, we demonstrated that 19% of analyzed patients survived 5 years with median of survival 17 months. The median PFS was 7 months. We demonstrated better outcomes than published results: median of OS patients ranged between 8.0 and 16.5 months, and median of PFS was between 4.1 and 6.1 months [16,17,18,19,20,21]. It is necessary to explore the factors that influenced long-term survival of patients with LCNEC. In our analysis, we found that treatment with radical intension, R0 resection margin, lower clinical stage, lymph node-negative, and size of tumour ≤4 cm led to significantly better survival prognosis (OS and PFS) (*p* < 0.05). The authors of various studies also looked for correlations between clinicopathologic factors and time of survival. Five-year OS of patients in different clinical stages in our study seems to be similar to that achieved by other authors, who estimated the median survival of patients with early-stage LCNECs between 19 and 40 months, with 5-year survival between 21% and 62%, based on retrospective studies of between 5 and 144 patients. Reported stage-for-stage 5-year survival rates in LCNEC are: stage I (33–62%), stage II (18–75%), stage III (8–45%), and 0% in patients with stage IV disease. Clinicopathologic factors that correlate with poor survival in published studies include advanced tumour stage, tumour size (greater than 3 cm), and male gender [16,22,23,24]. Naidoo et al. [15] evaluated the median overall survival at level 10.2 months (95% CI, 8.6–16.4 months) for the stage IV LCNEC cohort. In the univariate model of our analysis, significant influence on OS was demonstrated for clinical stage, lymph nodes status, size of tumour, intention of treatment (*p* < 0.001), and extent of resection (*p* = 0.02). We figured out the independent factors that influenced OS and PFS, like clinical stage and resection margin R0 vs. R1-2. The studies that have been published recently included 2594 LCNEC cases that revealed that gender, age, TNM stage, T stage, N stage, and M stage were all independent prognostic factors [25]. Cao et al. [26] also determined that tumour size and TNM stage were associated with survival in patients with pulmonary LCNEC in univariate analysis, but they discovered that older age at diagnosis (≥65 years) was an independent prognostic risk factor for OS; however, we did not achieve such a result. The multivariate analysis [16] that included 2368 LCNEC cases did not prove the association between tumour grade and LCNEC survival. Deng et al. [27] demonstrated that 5-year OS of LCNEC patients (*n* = 2097) was 16.7% with a median 11 months. Five-year OS was 43.9%, 24.1%, 12.7%, and 2.6% (*p* < 0.001) in stages I, II, III, and IV disease, respectively, and the median OS was 44, 19, 14, and 6 months in stages I, II, III, and IV disease, respectively. The results of multivariate analysis indicated that advanced tumour stage, advanced nodal stage, not undergoing surgery, and not undergoing chemotherapy were independent adverse indicators of OS. In addition, patients aged ≥65 years and male sex were also the independent adverse indicators related to OS.

A retrospective analysis of 144 surgical cases [28] showed that 42.5% patients survived 5 years, 52% for stage I, 59% for stage II, and 20% for stage III. A trend towards a better outcome was associated with preoperative or postoperative chemotherapy in stage I disease compared to no chemotherapy. In a study that included 197 patients who underwent surgery (without patients in stage IV), high-grade neuroendocrine carcinoma (HGNEC - LCNEC and SCLC) authors demonstrated that perioperative chemotherapy significantly improved the 5 year OS rates of HGNEC patients [29].

The multivariate analysis revealed that perioperative chemotherapy, lobectomy and lymph-node-negative were independently associated with survival [29]. The basic method of treatment of patients with early stage of cancer is resection (lobectomy is used most frequently; pneumonectomy with mediastinal lymph node dissection is less frequent). The criteria of patient’s qualification to surgery treatment and neoadjuvant chemotherapy are the same as in the case of non-small-cell lung cancer. For primary lung neuroendocrine neoplasms, LCNEC and LCNEC combined are characterized by a high risk of recurrence after surgery, even in CS I. This is the reason that adjuvant chemotherapy is recommended in all stages, including early clinical stages, of cancer advancement after surgery. Although the treatment of LCNEC is not clearly established and there are some difficulties such as rare occurrence and the retrospective character of studies, the authors investigated treatment factors that could correlate with survival. Some authors [16,26] revealed that surgery, radiation, and chemotherapy were associated with survival in patients with pulmonary LCNEC. In our study, method of radical treatment (surgery alone vs. resection with neoadjuvant/adjuvant treatment vs. radiochemotherapy) and type of chemotherapy (SCLC chemotherapy vs. NSCLC chemotherapy schedule) did not influence OS or PFS. There are limited published data regarding the clinical course and treatment of patients with advanced LCNEC. The National Cancer Control Network (NCCN) recommends treatment according to non-small-cell lung cancer (NSCLC) guidelines; however, LCNECs are frequently treated with the same chemotherapeutic regimens used for SCLC, given that they are both high-grade neuroendocrine neoplasms. While response rates of approximately 50% and up to 80% have been demonstrated in prospective studies utilizing platinum-based/etoposide in patients with extensive-stage SCLC (ED-SCLC), current reports suggest that LCNEC are substantially less chemo-responsive to this regimen. We are unaware of any randomized studies investigating how patients with advanced LCNECs should be optimally treated [18,20,30,31,32,33,34,35,36]. Gu et al. [25] demonstrated that surgery benefited stage I, II, and III LCNEC patients’ prognoses. The combination treatment including surgery and chemotherapy was the optimal treatment for stage I, II, and III LCENC patients. LCNEC is an aggressive tumour with a high rate of recurrence even after complete surgical resection in its early stage [37]; therefore, surgery alone could be insufficient to treat patients with LCNEC, and adjuvant treatment such as chemotherapy or radiation may be necessary. Chemoradiation achieved better overall response rate than chemotherapy alone [38]. The study conducted by Gu [25] did not admit that chemoradiation provides a benefit for stage I, II, and III surgery patients’ prognoses. The schedule of chemotherapy is still not established, independently of clinical stage of LCNEC. Patients are treated according to small-cell-lung cancer (SCLC) schedule of treatment (platinum + etoposide) or NSCLC schedule (platinum + gemcitabine vs. vinorelbine vs. paclitaxel).

In Europe and Japan, neoadjuvant, adjuvant and palliative chemotherapy recommended for LCNEC/combined LCNEC patients’ treatment is like typical for SCLC, but in USA, a more preferred schedule is like that of NSCLC. The rarity of this cancer results in a lack of randomized clinical trials for histopathologically confirmed LCNEC and no standards in combined, neoadjuvant, adjuvant, and palliative therapy, and the role of radiotherapy including elective radiotherapy on the central nervous system (CNS) according to the treatment protocol of SCLC has not been established. Sun et al. [18] compared efficacy of first-line chemotherapy for large-cell neuroendocrine carcinoma in the SCLC and NSCLC regimen groups, and the median progression-free survival times were 6.1 vs. 4.9 months, respectively, and the median overall survival was 16.5 vs. 9.2 months. Other authors [39] analyzed patients with the diagnosis of stage IV LCNEC and showed that NSCLC-t chemotherapy (first-line platinum-based combined chemotherapy, including gemcitabine, docetaxel, paclitaxel, or vinorelbine) improved overall survival in comparison to NSCLC-pt (with pemetrexed treatment only) and SCLC-t chemotherapy (consisting of etoposide chemotherapy). In our study, we did not achieve a significant difference in patients’ outcome (OS, PFS) between those treated with chemotherapy for SCLC vs. NSCLC.

## 5. Conclusions

Patients with LCNEC diagnosis were characterized by poor prognosis of median 7 and 17 months, respectively for PFS and OS. Independent prognostic factors influencing PFS were clinical stage (CS) and resection margin R0 vs. R1-2. 

## Figures and Tables

**Table 1:** Inclusion criteria.

Inclusion Criteria:	
1.	≥18 years old
2.	Caucasian race
3.	LCNEC or combined type LCNEC diagnosis
4.	The stage of cancer development was determined using UICC TNM classification of Malignant Tumors—8th Edition
5.	Diagnosis in clinical stage I-IV
6.	Patients without prior treatment
7.	Patients with generalized, unresectable of LCNEC before, during and after palliative treatment
8.	Patients with generalized, unresectable LCNEC treated only symptomatically
9.	Patients with locally advanced, unresectable LCNEC, combined LCNEC before, during and after radical treatment
10.	Patients with locally advanced, resectable LCNEC, combined LCNEC before, during and after treatment independently on type of treatment

**Table 2:** Characteristic of patients.

All		*N* = 132	%
Age [years] (range: 36–86, mean 64.1 ± 9.1)			
	≤64	66	50
	>64	66	50
Gender			
	women	47	36
	men	85	64
Histopathology			
	LCNEC	124	94
	combined LCNEC	8	6
Clinical stage			
	I	22	17
	II	19	14
	IIIA	22	17
	IIIB	13	10
	IIIC	3	2
	IV	53	40
Lymph nodes status			
	N0	38	29
	N1	17	13
	N2	56	42
	N3	21	16
Ki67			
	≤55	31	23
	>55	71	54
	no data	30	23
Localization			
	left lung	60	45
	right lung	72	55
Intention to treat			
	radical treatment	67	51
	palliative treatment	41	31
	symptomatic treatment	24	18
Type of radical treatment			
	alone surgery	30	45
	surgery with neo- or adjuvant chemotherapy or radiochemotherapy or with adjuvant alone radiotherapy	30	45
	radiochemotherapy	7	10
Radicality of surgical resection			
	R0	43	72
	R1-2	17	28
Type of palliative treatment			
	resection and palliative chemotherapy	3	7
	palliative chemotherapy	38	93
Size of tumour [mm] (range: 7–136, mean 52.2 ± 26.5)			
	≤4 cm	50	38
	>4 cm	82	62
Size of tumour			
	≤3 cm	34	26
	>3–5 cm	38	29
	>5–7 cm	30	22.5
	>7 cm	30	22.5
Type of chemotherapy			
	SCLC—chemotherapy	55	71.4
	NSCLC—chemotherapy	20	26
	other	2	2.6

±standard deviation. LCNEC—large-cell neuroendocrine lung cancer; combined LCNEC is LCNEC with components of adenocarcinoma, squamous cell carcinoma, or spindle cell carcinoma, and/or giant cell carcinoma. N0—no lymph node metastasis; N1—metastasis in ipsilateral peribronchial and/or hilar lymph nodes and intrapulmonary nodes; N2—metastasis in ipsilateral mediastinal and/or subcarinal lymph node(s); N3—metastasis in contralateral mediastinal or hilar, ipsilateral/contralateral scalene/supraclavicular lymph node(s). Ki67—a proliferation marker to measure the growth fraction of cells in human tumours. R0—negative margin, no tumour at the margin; R1—microscopic positive margin, tumour identified microscopically at the margin; R2—macroscopic positive margin, tumour identified grossly at the margin. SCLC (small-cell lung cancer)—type chemotherapy (platinum/carboplatinum-etoposide; PE/KE schedule); NSCLC (non-small-cell lung cancer)—type chemotherapy (platinum-vinorelbine; PN schedule).

**Table 3:** Differences in overall survival and progression-free survival of LCNEC cancer patients due to prognostic factors.

		Overall Survval (OS)				Progression-Free Survival (PFS)			
Variables		5-Year (%)	Median OS (95% CI Months)		Log-Rank Test	5-Year (%)	Median PFS (95% CI Months)		Log-Rank Test
All		19	17	(9.0–36.2)	*p*	13	7	(3.0–15.0)	*p*
Age									
	≤64	25	19	(10.0–52.5)	0.11	18	7	(4.0–25.2)	0.16
	>64	12	17	(7.0–28.7)		5	6	(0.5–12.0)	
Gender									
	women	24	22	(9.3–51.8)	0.29	13	12	(3.0–28.1)	0.33
	men	17	16	(7.0–33.1)		15	6	(3.0–12.0)	
Clinical stage									
	I	63	105	(25.6–not reached)	<0.001 *	49	54	(6.0-not reached)	<0.001 **
	II	25	28	(14.5–53.9)		-	27	(8.5–40.5)	
	IIIA	21	31	(15.6–51.6)		14	12	(6.0–21.0)	
	IIIB	15	19	(11.0–30.2)		-	6	(4.0–12.6)	
	IIIC	-	13	(9.0–16.0)		-	5	(4.0–6.0)	
	IV	-	8	(3.0–16.0)		-	3	(0.0–7.0)	
Lymph nodes status									
	N0	54	64	(21.9–not reached)	<0.001 ^#^	41	42	(7.0-not reached)	<0.001 ^##^
	N1	9	23	(14.0–36.8)		12	11	(6.3–24.3)	
	N2	6	13	(6.9–28.4)		-	4	(0.0–10.0)	
	N3	-	9	(3.3–16.0)		-	3	(0.0–6.0)	
Lymph nodes status									
	N+	6	13	(7.0–25.8)	<0.001	3	5	(1.3–10.6)	<0.001
	N−	54	64	(21.9–not reached)		41	42	(7.0-not reached)	
Ki67									
	≤55	22	22	(11.2–51.6)	0.56	21	10	(3.8–38.5)	0.66
	>55	22	18	(7.0–48.5)		16	7	(3.0–28.2)	
Localization									
	left lung	18	22	(13.0–43.7)	0.13	12	9	(5.0–17.0)	0.26
	right lung	20	15	(7.0–36.3)		15	4	(2.0–13.0)	
Intention to treat									
	radical treatment	39	48	(19.0–87.5)	<0.001 ^	26	16	(6.0–59.0)	<0.001 ^^
	palliative treatment	-	12	(9.0–17.0)		-	4	(3.0–9.1)	
	symptomatic treatment	-	3	(1.0–7.0)		-	0	(0.0–3.0)	
Type of radical treatment									
	Surgery alone	23	37	(14.2–54.0)	0.35	22	11	(4.5–47.9)	
	Surgery with neo-or adjuvant chemotherapy or radiochemotherapy or with alone adjuvant radiotherapy	45	48	(17.2–59.2)		29	17	(8.6-not reached)	0.28
	Radiochemotherapy								
	Surgery alone	23	37	(14.2–54.0)	0.26	22	11	(4.5–47.9)	
	Surgery with neo-or adjuvant chemotherapy or radiochemotherapy or with adjuvant radiotherapy alone	46	58	(22.1–not reached)		36	31	(9.0-not reached)	0.16
	Surgery alone	23	37	(14.2–54.0)	0.87	22	11	(4.5–47.9)	0.69
	Radiochemotherapy	36	35	(16.8–44.9)		-	10	(5.5–21.8)	
**Radicality of surgical resection**									
	R0	47	58	(22.0–not reached)	0.09	40	31	(7.0-not reached)	0.02
	R1-2	15	27	(11.8–39.3)		7	9	(3.0–18.7)	
**Type of palliative treatment**									
	Resection and palliative chemotherapy	-	6	(6.0–22.0)	0.94	-	13	(2.0–13.0)	0.50
	Palliative chemotherapy	-	12	(9.0–17.0)		-	4	(3.0–9.0)	
**Size of tumour**									
	≤4 cm	30	29	(14.9–81.7)	<0.001	22	10	(5.0–46.1)	0.002
	>4 cm	12	13	(6.4–28.0)		8	5	(1.3–12.0)	
**Size of tumour**									
	≤3 cm	36	48	(16.1–not reached)	<0.001 ^§^	24	11	(6.0–52.0)	<0.001 ^§§^
	>3–5 cm	16	19	(7.3–36.8)		13	9	(3.0–18.4)	
	>5–7 cm	19	16	(6.4–31.2)		13	7	(1.0–15.6)	
	>7 cm	6	12	(3.5–17.0)		-	4	(0.0–8.4)	
**Type of chemotherapy**									
	SCLC—chemotherapy	22	21	(11.4–40.6)	0.93	13	9	(4.0–16.3)	0.86
	NSCLC—chemotherapy	27	17	(12.0–not reached)		16	9	(4.0–10.7)	

* I vs. II (*p* = 0.02); I vs. IIIA (*p* = 0.02); I vs. IIIB (*p* = 0.003); I vs. IIIC (*p* = 0.01); I vs. IV (*p* < 0.001); II vs. IIIA (*p* > 0.05); II vs. IIIB (*p* > 0.05); II vs. IIIC (*p* > 0.05); II vs. IV (*p* < 0.001); IIIA vs. IV (*p* < 0.001); IIIB vs. IV (*p* < 0.001); IIIC vs. IV (*p* = 0.94). ^#^ N0 vs. 1 (*p* = 0.02); N0 vs. 2 (*p* < 0.001); N0 vs. 3 (*p* < 0.001); N1 vs. 2 (*p* = 0.05); N1 vs. 3 (*p* < 0.001); N2 vs. 3 (*p* = 0.03). ^ rad. vs. palliat. (*p* < 0.001); rad. vs. symp. (*p* < 0.001); palliat. vs. symp. (*p* = 0.005). ^§^ ≤3 vs. >3–5 cm (*p* = 0.002); ≤3 vs. >5–7 cm (*p* = 0.004); ≤3 vs. >7 cm (*p* < 0.001); >3–5 vs. >5–7 cm (*p* = 0.75); >3–5 vs. >7 cm (*p* = 0.04); >5–7 vs. >7 cm (*p* = 0.12). ** I vs. II (*p* = 0.32); I vs. IIIA (*p* = 0.03); I vs. IIIB, 0.02; I vs. IIIC, 0.07; I vs. IV, <0.001; II vs. IIIA, 0.27; II vs. IIIB, 0.08; II vs. IIIC, 0.07; II vs. IV, <0.001; IIIA vs. IV, <0.001; IIIB vs. IV, <0.001; IIIC vs. IV, 0.99. ^##^ N0 vs. 1 (*p* = 0.07); N0 vs. 2 (*p* < 0.001); N0 vs. 3 (*p* < 0.001); N1 vs. 2 (*p* = 0.009); N1 vs. 3 (*p* < 0.001); N2 vs. 3 (*p* = 0.12). ^^ rad. vs. palliat. (*p* < 0.001); rad. vs. symp. (*p* < 0.001); palliat. vs. symp. (*p* = 0.03). ^§§^ ≤3 vs. >3–5 cm (*p* = 0.046); ≤3 vs. >5–7 cm (*p* = 0.07); ≤3 vs. >7 cm (*p* < 0.001); >3–5 vs. >5–7 cm (*p* = 0.79); >3–5 vs. >7 cm (*p* = 0.01); >5–7 vs. >7 cm (*p* = 0.04).

**Table 4:** Univariate Cox regression.

		Overall Survval (OS)			Progression-Free Survival (PFS)		
Variables		HR (95% CI)		*p*	HR (95% CI)		*p*
Age	≤64	1.00	Reference		1.00	Reference	
	>64	1.40	(0.93–2.10)	0.11	1.31	(0.89–1.92)	0.17
Gender	women	1.00	Reference		1.00	Reference	
	men	>1.24	>(0.82–1.89)	0.31	>1.20	>(0.81–1.80)	0.36
Clinical stage	I	1.00	Reference		1.00	Reference	
	II	3.20	(1.19–8.66)	0.02	1.58	(0.68–3.69)	0.29
	IIIA	3.12	(1.20–8.12)	0.02	2.33	(1.08–5.04)	0.03
	IIIB	5.44	(1.96–15.13)	0.001	3.53	(1.46–8.56)	0.005
	IIIC	14.40	(3.44–60.27)	<0.001	7.95	(2.10–30.12)	0.002
	IV	15.90	(6.48–39.02)	<0.001	8.59	(4.15–17.82)	<0.001
Lymph nodes status	N0	1.00	Reference		1.00	Reference	
	N1	2.52	(1.19–5.31)	0.02	0.86	(0.38–1.93)	0.22
	N2	4.33	(2.40–7.82)	<0.001	3.85	(2.27–6.54)	0.007
	N3	9.42	(4.61–19.28)	<0.001	5.96	(3.11–11.40)	<0.001
Ki67	≤55	1.00	Reference		1.00	Reference	
	>55	1.16	(0.69–1.93)	0.57	1.11	(0.68–1.79)	0.68
Localization	left lung	1.00	Reference		1.00	Reference	
	right lung	1.35	(0.90–2.02)	0.14	1.23	(0.84–1.80)	0.29
Intention to treat	radical treatment	1.00	Reference		1.00	Reference	
	palliative treatment	4.88	(2.93–8.15)	<0.001	3.58	(2.21–5.80)	0.13
	symptomatic treatment	11.10	(6.15–20.03)	<0.001	6.73	(3.83–11.82)	<0.001
Radicality of surgical resection	R0	1.00	Reference		1.00	Reference	
	R1-2	2.28	(1.12–4.63)	0.02	2.43	(1.30–4.54)	0.005
Size of tumour	≤3 cm	1.00	Reference		1.00	Reference	
	>3–5 cm	1.27	(0.66–2.45)	0.75	0.90	(0.49–1.65)	0.77
	>5–7 cm	2.63	(1.39–4.96)	0.45	1.65	(0.93–2.93)	0.94
	>7 cm	4.35	(2.29–8.24)	<0.001	2.98	(1.69–5.26)	<0.001

**Table 5:** Multivariate Cox regression.

		Overall Survival (OS)			Progression-Free Survival (PFS)		
Variables		HR (95% CI)		*p*	HR (95% CI)		*p*
Clinical stage	I	1.00	Reference		1.00	Reference	
	II	4.00	(1.43–11.16)	0.008	2.08	(0.86–5.01)	0.10
	IIIA	1.81	(0.55–5.98)	0.33	1.12	(0.43–2.92)	0.81
	IIIB	3.50	(0.69–17.70)	0.13	1.19	(0.24–5.96)	0.83
	IIIC	-	-	-	-	-	-
	IV	1.90	(0.23–15.51)	0.55	10.53	(2.29–48.38)	0.002
Intention to treat	radical treatment	1.00	Reference		1.00	Reference	
	palliative treatment	2.27	(0.23–22.02)	0.48	0.34	(0.05–2.11)	0.24
	symptomatic treatment	1.27	(0.14–11.62)	0. 83	0.44	(0.05–4.01)	0.47
Radicality of surgical resection	R0	1.00	Reference		1.00	Reference	
	R1-2	2.38	(0.79–7.17)	0.12	3.06	(1.26–7.42)	0.01

## Data Availability

Raw data is available from the author upon request.
